# Neural field simulator: two-dimensional spatio-temporal dynamics involving finite transmission speed

**DOI:** 10.3389/fninf.2015.00025

**Published:** 2015-10-20

**Authors:** Eric J. Nichols, Axel Hutt

**Affiliations:** Team Neurosys, Loria, Centre National de la Recherche Scientifique, INRIA, UMR no. 7503, Université de LorraineNancy, France

**Keywords:** neural field, numerical simulation, delay, pattern formation

## Abstract

Neural Field models (NFM) play an important role in the understanding of neural population dynamics on a mesoscopic spatial and temporal scale. Their numerical simulation is an essential element in the analysis of their spatio-temporal dynamics. The simulation tool described in this work considers scalar spatially homogeneous neural fields taking into account a finite axonal transmission speed and synaptic temporal derivatives of first and second order. A text-based interface offers complete control of field parameters and several approaches are used to accelerate simulations. A graphical output utilizes video hardware acceleration to display running output with reduced computational hindrance compared to simulators that are exclusively software-based. Diverse applications of the tool demonstrate breather oscillations, static and dynamic Turing patterns and activity spreading with finite propagation speed. The simulator is open source to allow tailoring of code and this is presented with an extension use case.

## 1. Introduction

The understanding of spatio-temporal electric activity in neural tissue is essential in the study of neurobiological phenomena. To achieve this, mesoscopic-scale models such as neural mass and neural fields (NFM) which describe the dynamics of a large population of neurons reflecting coarse-grained properties of single neurons (Wilson and Cowan, 1973; Deco et al., 2008; Bressloff, 2012; Hutt and Buhry, 2014) play an important role. NFMs serve as a good description of the dynamic source of Local Field Potentials and encephalographic data (Nunez, 1974, 2000; Wright and Kydd, 1992; Wright and Liley, 1994; Jirsa et al., 2002; Nunez and Srinivasan, 2006; Coombes et al., 2014). They allow to consider diverse single neuron features that may tune neural population dynamics, such as somatic (Molaee-Ardekani et al., 2007) and synaptic adaptation (Coombes and Owen, 2005; Kilpatrick and Bressloff, 2010), extra-synaptic receptor dynamics (Hashemi et al., 2014; Hutt and Buhry, 2014) or finite axonal transmission speed (Jirsa and Haken, 1996; Pinto and Ermentrout, 2001; Hutt et al., 2003, 2008; Coombes, 2005; Faye and Faugeras, 2010; Veltz and Faugeras, 2011, 2013). All these applications make NFMs valuable in order to understand spatio-temporal dynamics of neural population activity.

Mathematical analysis and the numerical integration of NFMs are complementary. The recent years have shown strong attention of research on the mathematical properties of NFMs, whereas the numerical simulation of NFM solutions has been less considered in research. Since NFMs generalize partial differential equations (Coombes et al., 2007; Hutt, 2007) while involving finite transmission delay interactions, they allow to study a large class of pattern forming systems, cf. Hutt (2007). In recent years, several software tools have been developed to simulate neural network dynamics. Examples for simulators for networks of spiking neurons are *BRIAN* (Stimberg et al., 2014) and *Neuron* (Carnevale and Hines, 2006). *The Virtual Brain* (Sanz Leon et al., 2013) allows to simulate networks of neural mass models to reproduce global brain activity. The simulation platform *DANA* (Rougier and Fix, 2012) simulates a hierarchy of coupled Dynamic Neural Fields which are decentralized, i.e., are updated numerically in time asynchronously (Rougier and Hutt, 2011). These latter software tools are powerful, general and highly adaptive to the framework of their neural network types. However, they do not provide the effective computation for the specific NFM given in Equation (1) which is a stochastic delayed integral-differential equation in two spatial dimensions. The tool presented here fills a gap in the landscape of neural simulator tools which are typically very general and adaptive and, hence, not efficient for NFM. A simulation tool for NFM allows to explore rapidly and in a user-friendly way the solution space of Equation (1) in order to reproduce numerically experimental spatio-temporal dynamics, e.g., to understand neuroimaging data (Friston et al., 2014; Pinotsis and Friston, 2014), retrieve neural sources and lateral connections (Pinotsis et al., 2013), and understand power spectra of electroencephalographic activity (Pinotsis et al., 2012). In addition, the tool promises to allow detection of new numerical solutions, cf. Section 3. The numerical analysis is non-trivial and challenging if NFMs become more complex, e.g., by involving complex dynamical features rendering the model high-dimensional or by considering delayed interactions. The present work considers a two-dimensional spatial embedding of neural populations similar to several previous studies (Laing, 2005; Owen et al., 2007) while taking into account finite axonal transmission speed (Hutt and Rougier, 2010, 2014). By virtue of its modularity, the simulator allows subsequent extensions with additional features, such as extra-synaptic receptor effects or several interacting populations.

The combination of finite axonal transmission speed and two-dimensional spatial embedding is challenging from a numerical simulation perspective due to the missing convolution structure (Hutt and Rougier, 2010, 2014) leading to long simulation durations. To overcome this problem, a numerical technique has been developed in recent years (Hutt and Rougier, 2010, 2014). Since future research in neural fields will investigate spatio-temporal dynamics involving finite axonal transmission speed, we have developed an open-source simulation toolbox that allows to gain spatio-temporal solutions of NFM models in two spatial dimensions, visualize them and save them, if necessary, as movies. We hope that the tool will provide an essential tool for the computational neuroscience community to advance the research field and the insight into the brain.

The simulator in this work obeys integral-differential equations of the type

(1) $$\begin{array}{l} \left(\eta \frac{\partial^{2}}{\partial t^{2}}+\gamma \frac{\partial }{\partial t}+1\right)\text{​​​​​​ }V(x,t)=I(x,t)+\int_{\Omega }\;K(x-y)\\ \;S\left[V\left(y,t-\frac{∥x-y∥}{c}\right)\right]{\text{d}}^{2}y \end{array}$$

with a two-dimensional square spatial domain Ω and periodic boundary condition. The mean neuron potential *V* ∈ R at location **x** ∈ Ω is evolved by the external stimulus *I*(*x, t*) ∈ R and the integral of the synaptic connectivity kernel *K* : R^2^ → R and population firing rate *S* ∈ R which depend on the distance between spatial locations **x** and **y** with a finite axonal transmission speed *c*. Equation (1) represents the core of most NFM in the sense that most NFMs consider extensions of this equation.

Motivation for the work arises from a need for a visualization tool that is useful to the largest number of NFM researchers, allows for the tailoring of code and has fast while visually appealing output. The simulator can operate on all major operating systems. Output of data in three dimensions is provided by PyOpenGL which brings the speed and graphical detail of low-level OpenGL to the agile Python language. It is open source, enabling modification of the simulator in any beneficial way.

## 2. Materials and methods

The cross-platform simulator is written in Python (version 2.7) and uses the NumPy library in consideration of its speed being close to the computational rate of the platform-dependent C language (Langtangen, 2006). The simulator can be downloaded^1^ in a package along with documentation^2^ describing its installation, running, features, and examples and the code is registered in ModelDB^3^.

The following Section 2.1 describes the comprehensive access to field parameters, the subsequent section details the techniques applied to accelerate the simulation and Section 2.3 discusses the 3D visualization.

### 2.1. Field parameters

A textual interface named values.py is provided in the root directory of the simulator code. It allows field values to be changed without knowledge of the inner workings of the simulator. For example, if η in Equation (1) is initialized as a non-zero number, a second order derivative is calculated to solve *V*. Conversely, the interface eliminates the knowledge requirement of the numerical implementation of the derivatives and all other underlying code implementations. The interface has additional benefits of easily modifying variables in one place without searching through the code. This implementation permits changing parameters easily and sharing code amongst others working with similar simulations by the exchange of a single file.

The most important aspect of a text-based interface from its user experience is its facilitation of novelty by allowing absolute control of all terms of Equation (1). For instance, matrix *I* can be defined in the interface with as many lines of Python code as necessary given the definition ends with an assignment (i.e., *I = …*). Assigning the first parameter in the values.py file, named showData, a value of 3 displays *I* in the simulator, which can be useful when refining its values. Time-varying spatio-temporal input is available in the interface by uncommenting and modifying the body of a function named *updateI* in any manner while maintaining that *I* is returned. Neural field investigations are thereby efficiently implemented with free choice over all the variables accessible through the interface while retaining the full performance.

### 2.2. Accelerated simulation

The simulator is advantageous in its acceleration of spatial and temporal integration. Multiple approaches are used to increase the simulation speed.

#### 2.2.1. Spatial and temporal integration

Equation (1) includes a spatial integral with a homogeneous kernel *K*. Please recall that homogeneous kernels just depend on the difference vector **x** − **y** between two spatial locations **x, y** including isotropic kernels, i.e., *K* = *K*(∥**x** − **y**∥), as a specific case. In the absence of the finite transmission delay term, this integral would represent a spatial convolution and would be solvable numerically efficiently by a Fast Fourier Transform (FFT) (Van Loan, 1991). For non-vanishing transmission delay, the convolution structure is less obvious and the FFT is not applicable directly. Nevertheless, it is possible to re-write the spatial integration to utilize a FFT in space (Owen et al., 2007; Hutt and Rougier, 2010, 2014) as

$$\begin{array}{l} \int_{\Omega }{\text{d}}^{2}y\;K(x-y)\;S\left[V\left(y,t-\frac{∥x-y∥}{c}\right)\right]=\int_{\Omega }{\text{d}}^{2}y\int_{0}^{\tau_{m}}\\ \;d\tau L(x-y,t-\tau )\;S\left[V\left(y,t-\tau \right)\right] \end{array}$$

with the maximum delay time τ_*m*_ and the spatio-temporal kernel function *L*(**x**, *t*) = *K*(**x**)δ(∥ **x** ∥ ∕*c* − *t*). We observe that the spatial summation represents an integration over delayed spatial rings, which are convolved spatially with the transfer function *S* in Equation (1). Introducing a regular rectangular spatial grid for spatial discretization, finite axonal speed *c* yields rings of width

(2) w=max(1, c·Δt·n/l)

delineated within the field, where *n* and *l* are the number of discretized spatial units and the length of the field, respectively, and Δ*t* is the finite integration time step. The Pythagorean theorem gives the maximum radius of the rings in the field $r=n∕\sqrt{2}$ over which the spatial integration is performed, which is applied to obtain the number of rings in a field as

(3) $$n_{rings}=1+\left\lfloor r/w\right\rfloor =1+\left\lfloor 1/\sqrt{2}c\Delta t\right\rfloor$$

defining the maximum delay to τ_*m*_ = *n*_*rings*_Δ*t*. The spatio-temporal kernel *L* is determined by the spatial kernel *K* and the axonal speed *c* (Hutt and Rougier, 2010, 2014).

Equation (1) involves distance-dependent delays which represent a specific type of distributed delays (Hutt and Lefebvre, in press). To this end, it is necessary to initialize the field variable *V* in an initial time interval and the toolbox allows the user to set the initial values arbitrarily. The external input *I* may be deterministic or stochastic and the user may choose it according to her needs, e.g., implementing spatial correlations in stochastic inputs. To integrate the evolution equation in time the user may choose between different integration methods for delay differential equations (Buckwar and Winkler, 2006, 2007). Standard methods discretize time regularly in steps of duration Δ*t* yielding results (Equations 2, 3). In the case of stochastic input, the toolbox includes numerical implementations of the delayed Euler-Maruyama method (Buckwar et al., 2008) and the stochastic version of the Runge-Kutta method for delayed differential equation (Carletti, 2006). For deterministic inputs, the equivalent deterministic methods are available.

If there is no modification to *K* and *c* during the simulation, then *L* is calculated once only before the start of the simulation while *S*(·) changes over time. The convolution of *L* and *S* is performed using a FFT what greatly increases the speed of the integral convolution compared to conventional integration. This can be understood easily recalling that the two-dimensional FFT needs to sum up $n^{2}\log_{2}^{2}\left(n\right)$ terms leading to summands of the total number of $N_{FFT}=n^{2}\log_{2}^{2}\left(n\right)n_{rings}$. In contrast, conventional integration sums up terms of number *n*^4^ for each delay time and hence the total number of computation $N_{conv}=n^{4}n_{rings}$, cf. Appendix I. Hence the FFT implementation speeds up the integration by a factor of

(4) $$f_{speedup}=\frac{N_{FFT}}{N_{conv}}=\frac{n^{2}}{\log_{2}^{2}(n)}$$

The axonal speed's implementation is described in detail in Hutt and Rougier (2010). It is important to note that other (conventional) numerical software tools not taking into account the convolution structure have to sum up *N*_*conv*_ terms in case of fully connected networks. For instance, this holds true for the simulation tools *BRIAN, Neuron* and *The Virtual Brain* (Sanz Leon et al., 2015) which have to memorize the history and advance the stored activity field *n*_*rings*_ times. We also note that the method proposed may be implemented in these simulation tools in the future since they also may consider spatially homogeneous neural fields as specific cases. The FFT-based method presented here computes the network interactions faster than these tools by *f*_*speedup*_ given in Equation (4). For instance, for a typical number of spatial grid intervals of *n* = 512 as used in the application showed in Section 3, we obtain the huge speed up factor of *f*_*speedup*_ = ≈3236.

#### 2.2.2. Self-writing code

The second approach to increase the simulation speed employs self-writing code to reduce the simulator's instruction set. The simulator writes and executes its own code to increase the efficiency of simulations and display only the user defined features. The simulation code is based on interface selections and is self-written by an initialization module at the onset of the program. The interface offers features, such as a second derivative calculation, *I* and *K* updates and added noise, that conditionally run during the simulation and are not performed over time if the user selects to view *V*, *I*, or *K* at *t* = 0. For example, the visual interface offers the choice of viewing the spatial kernel *K* for its design and visualization. Only the code that initializes and displays *K* is written to the executing module if this choice is selected. The self-writing code is also favorable when the full simulation is run with calculations executed unconditionally. The result is very efficient code that is changed with every modification to the interface.

#### 2.2.3. Implementation on GPUs

The third approach parallelizes the output calculations on the graphics processing unit (GPU). The displayed matrix is put onto the running system's GPU for hardware acceleration of the visualization. Vertex buffer objects improve visualization throughput by uploading vertices to video device memory where vertex and fragment shaders transform and write neural field data in parallel to the framebuffer for display. The simulator also avoids the CPU to GPU information transfer bottleneck by its storage of data on the video device memory. This is accomplished with the *OpenGL Shading Language* that is used through PyOpenGL to achieve a better visual description of information than is provided in other tools. A background on PyOpenGL and its comparison to other visualization libraries can be found in Rossant and Harris (2013).

#### 2.2.4. Optimal visualization rate

The fourth approach to accelerate simulations is to display field matrices at a rate optimized for continuous visualization perception. Two images are perceived simultaneously when there is an interval of less than 30 ms between them (Wertheimer et al., 2012). The simulator takes advantage of the temporal lag in biological visual perception by stopping the numerical calculations to submit the field data to the GPU once within every 30 ms. This allows for the numerical part of the simulations to continue with fewer stoppages, resulting in faster simulations.

### 2.3. 3D visualization

The open source and cross-platform *show3D* library was written for the Neural Field Simulator to display field information. The library's visualization of neural fields expands two dimensional neural field data into a third dimension to better observe the differences in field locations. This is achieved by raising every value in the 2D spatial plane to a third dimension position [*x*, *y*] ↦ [*z*] relative to other 2D field values.

**Figure 1:**
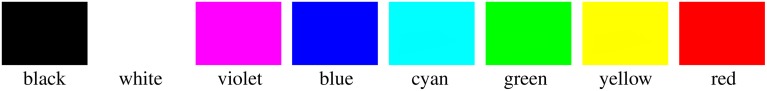
**Selection of colors that can be applied to the background and ranges of the plotted matrix**.

Color values are efficiently manipulated with the keyboard keys shown in Appendix II (Table A1). There is a selection of 8 colors, cf. Figure 1, available for the background, minimum, middle, and maximum graph values.

Intermediate color transformations are encoded in a dictionary containing 8^2^ unique 3 element vectors, each representing red, green, and blue colors. The appropriate color transformation vector is uploaded to the GPU where the vector elements represent one mutually inclusive index of [0, 1, *Z*, 1-*Z*] with Z axis locations ∈ **R** ∣ 0 < *Z* < 1. Each location on the Z axis is subsequently colored in parallel by the GPU with the appropriate shade. Graph value colors are interpolated with two choices of ranges: [minimum, maximum] and [minimum, middle], [middle, maximum] graph value colors. Different depths of the graph can be highlighted by raising or lowering the ranges of colors.

**Figure 2:**
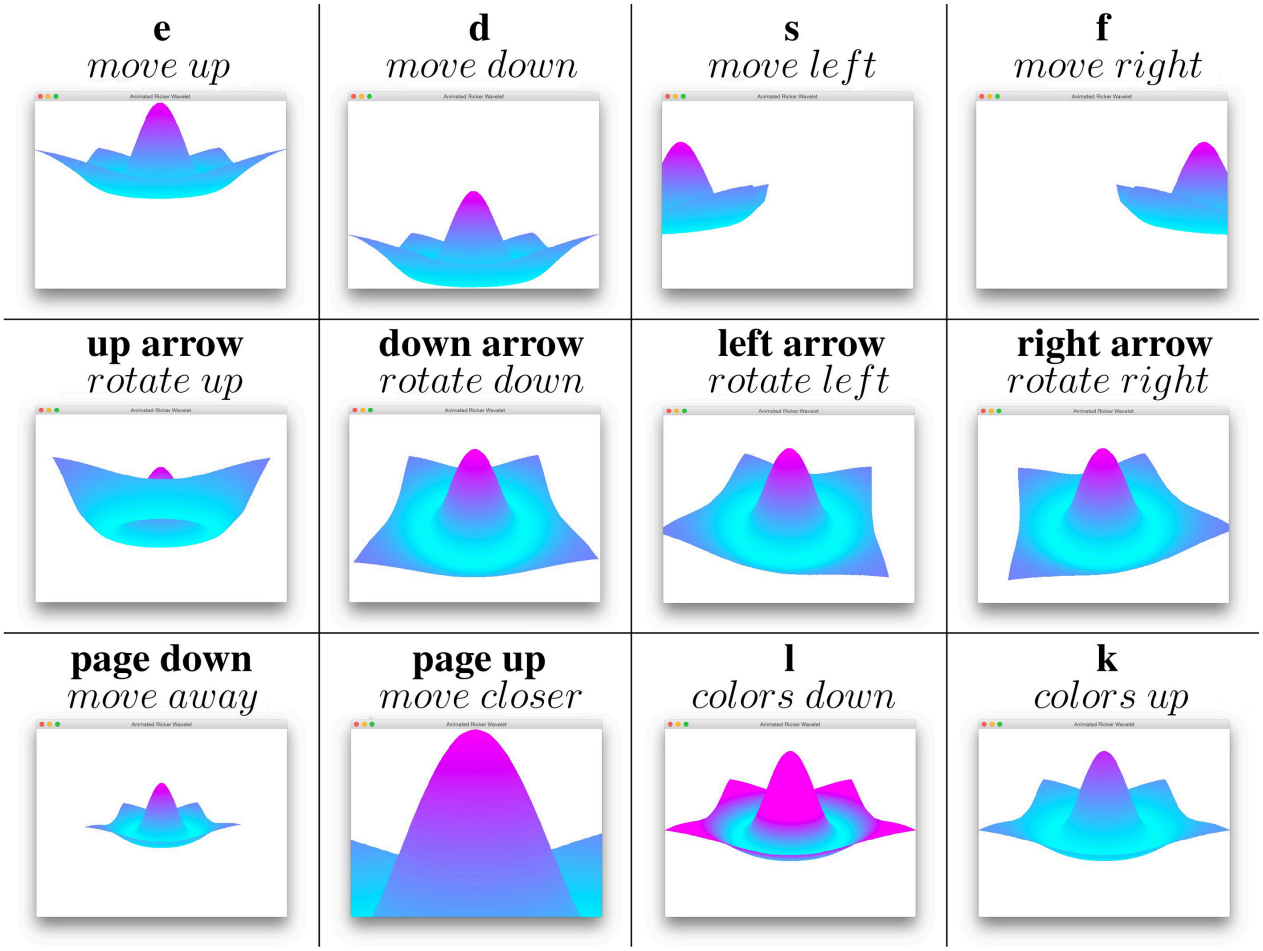
**Keyboard keys and their corresponding movements**.

Scrolling the mouse rotates neural fields in the direction of the mouse and the keyboard is used to move fields in various ways, cf. Figure 2.

Images and videos of simulations are saved, respectively in .png and .mp4 formats by using the keyboard keys in Appendix II. Visualization parameters are saved by the library after every simulation to reduce graphical adjustments during subsequent simulations of neural fields.

The show3D graphical visualization library is not limited to neural field data. Every two dimensional NumPy matrix can be displayed in 3 dimensions using the show3D library. Documentation for the show3D library's use, including a tutorial and code API, is online^4^ and packaged with example code along with the library^5^. However, there is no requirement for the library's separate download for use with the simulator because it is integrated into the Neural Field Simulator.

## 3. Applications

The simulator can be used to analyze spatio-temporal neural field dynamics. The simulator's open source code allows modifications and extensions to be added to the code. The subsequent sections describe few of these possible applications.

Introducing finite axonal transmission speed in neural fields substantially slows numerical computation. However, to omit finite transmission speed is to neglect biological physiology (Idiart and Abbott, 1993). Hutt and Rougier (2010) have suggested to implement finite axonal transmission speed in a computationally efficient manner that is utilized by the simulator. Numerically, the speed is infinite if $c\geq l∕\sqrt{2}\Delta t$ and there is increasing delay as *c* decreases.

### 3.1. Breather

Breather oscillations have been reported in theory (Folias and Bressloff, 2005; Hutt and Rougier, 2010) and experiments (Wang, 2010). As shown in Hutt and Rougier (2010), breathers are solutions of Equation (1) for finite axonal transmission speeds. They can be obtained and visualized in the simulator by assuming a temporally constant external input *I* in Equation (1). For a Gaussian-shape input with its apex at the center of the field, one overwrites the *I* variable section in the values.py file as

$$\begin{array}{l} \text{sigma}\;=\;5.65685425\\ \text{I }=\;20*\text{np}.\text{exp}\left(-\text{x}**2/\text{sigma}**2\right)\;/\;\left(\text{sigma}**2*\text{np}.\text{pi}\right)\; \end{array}$$

and change the showData variable assignment near the beginning of the values.py file to

**Figure 3:**
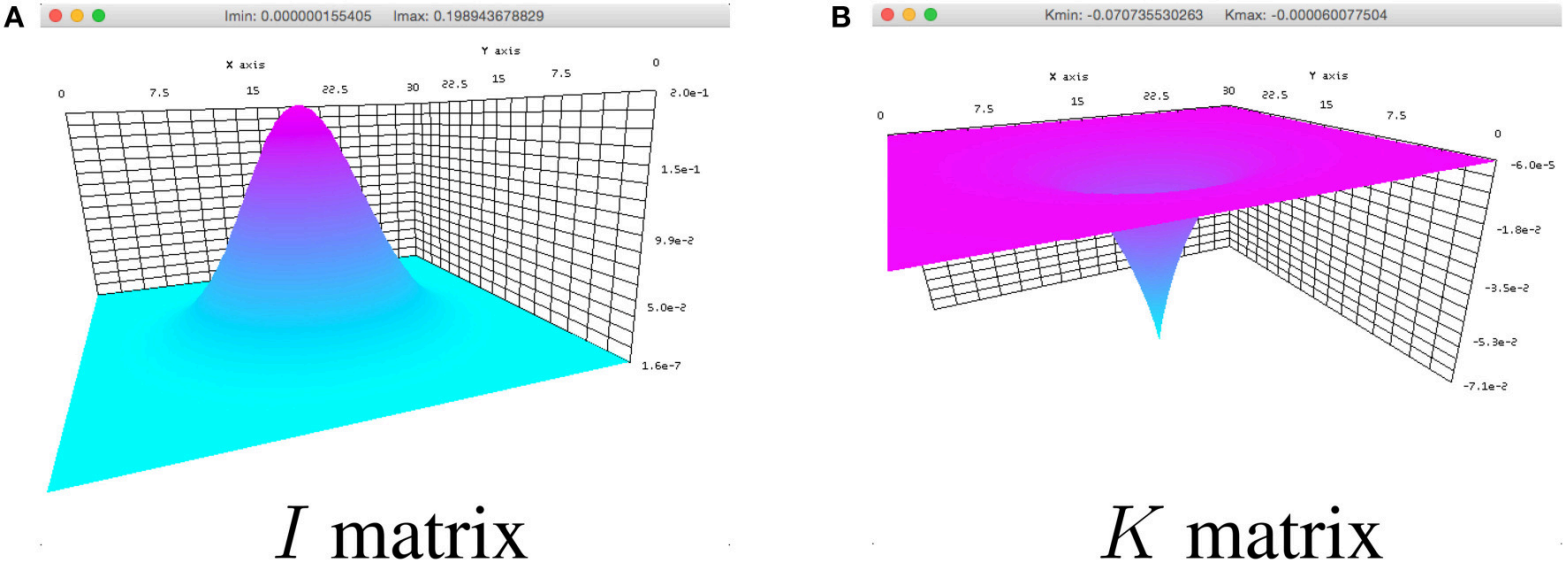
**Breather parameters plotted in the simulator for (A) *I* and (B) *K* in Equation (1)**.

showData = 3

to show the input *I* in the simulation. In the definition of *I*, the space variable *x* ∈ R^2^ is defined to cover the spatial domain Ω (not shown in the code snippet). A field input similar to Figure 3A can be seen when the simulator is run.

An inhibitory synaptic connectivity kernel, *K* in Equation (1), can be implemented for the breather and viewed by changing the *showData* and *K* variables in the values.py file to

$$\begin{array}{l} \text{showData}\;=\;4\\ \text{K}\;=\;-4*\text{np}.\text{exp}\left(-\text{x}/3\right)\;/\;\left(18*\text{np}.\text{pi}\right) \end{array}$$

and running the simulator. Here, *x* ∈ R^2^. An inhibitory synaptic kernel similar to the one in Figure 3B can be subsequently viewed.

After overwriting *I* and *K* as noted above, replace the following variables and function in the values.py file with the values below:

$$\begin{array}{l} \text{showData}\;=\;1\\ \text{endTime}\;=\;-1\\ \text{dt}\;=\;0.002\\ \text{gamma}\;=\;1.0\\ \text{eta}\;=\;0.0\\ \text{c}\;=\;500.0\\ \text{l}\;=\;30.0\\ \text{n}\;=\;512\\ \text{V}0\;=\;\text{np}.\text{zeros}\left(\left(\text{n},\text{n}\right)\right)\\ \text{noiseVcont}\;=\;\text{np}.\text{exp}\left(-\left(\text{a}**2\;/\;32.0+\text{b}**2/32.0\right)\right)\;/\;\\ \;\left(\text{np}.\text{pi}*32\right)*0.1*\text{np}.\text{sqrt}\left(\text{dt}\right)\\ \text{def}\;\text{updateS}\left(\text{V}\right):\\ \;\text{return}\;1.0\;/\;\left(1+\text{np}.\text{exp}\left(-10000*\left(\text{V}-0.005\right)\right)\right)\; \end{array}$$

**Figure 4:**
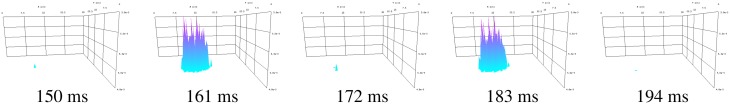
**Two cycles of the breather oscillations**.

Spatially localized breather oscillations are replicated by running the program. Figure 4 shows two cycles of the oscillations after setting the minimum and maximum z axis values by typing

$$\begin{array}{l} \text{n }0.00\text{48 }\left[\text{Enter key}\right]\\ \text{y }0.00\text{58 }\left[\text{Enter key}\right] \end{array}$$

after running the program.

### 3.2. Turing patterns

Turing patterns (Turing, 1952) have been reported in neural field models (Atay and Hutt, 2006; Elvin et al., 2009; Steyn-Ross et al., 2010). The Neural Field Simulator is able to compute and display noisy neural field activity evolving into Turing patterns.

Static Turing patterns emerge from noisy initial conditions with the following interface properties:

$$\begin{array}{l} \;\text{showData}\;=\;1\\ \text{endTime}\;=\;10\\ \text{dt}\;=\;0.01\\ \text{gamma}\;=\;1.0\\ \text{eta}\;=\;0.0\\ \text{c}\;=\;6364.0\\ \text{l}\;=\;90.0\;\\ \text{n}\;=\;512\\ \text{V}0\;=\;\text{np}.\text{ones}\left(\left(\text{n},\text{n}\right)\right)*5.\text{4 }+\text{np}.\text{random}.\text{normal}\\ \;\left(0,0.1,\left(\text{n},\text{n}\right)\right)\\ \text{noiseVcont}\;=\;\text{None}\\ \;\text{I}\;=\;\text{np}.\text{zeros}\left(\left(\text{n},\text{n}\right)\right)\\ \text{lins}\;=\;\text{np}.\text{linspace}\left(0,\;9*\text{np}.\text{pi},\;\text{n}\right)\;*-1\\ \text{K}\;=\;\text{np}.\text{zeros}\left(\left(\text{n},\;\text{n}\right)\right)\\ \text{for}\;\text{i}\;\text{in}\;\text{range}\left(\text{n}\right):\;\\ \;\text{K}\left[:,\text{i}\right]\;=\;\text{np}.\text{sin}\left(\text{lins}\left[\text{I}\right]\right)/150\;\\ \text{for}\;\text{i}\;\text{in}\;\text{range}\left(\text{n}\right):\;\\ \;\text{K}\left[\text{i}\right]\;-=\;\text{np}.\text{sin}\left(\text{lins}\left[\text{i}\right]\right)/200\;\\ \text{def}\;\text{updateS}\left(\text{V}\right):\\ \;\text{return}\;2.0\;/\;\left(1+\text{np}.\text{exp}\left(-1.24*\left(\text{V}-3.0\right)\right)\right) \end{array}$$

**Figure 5:**
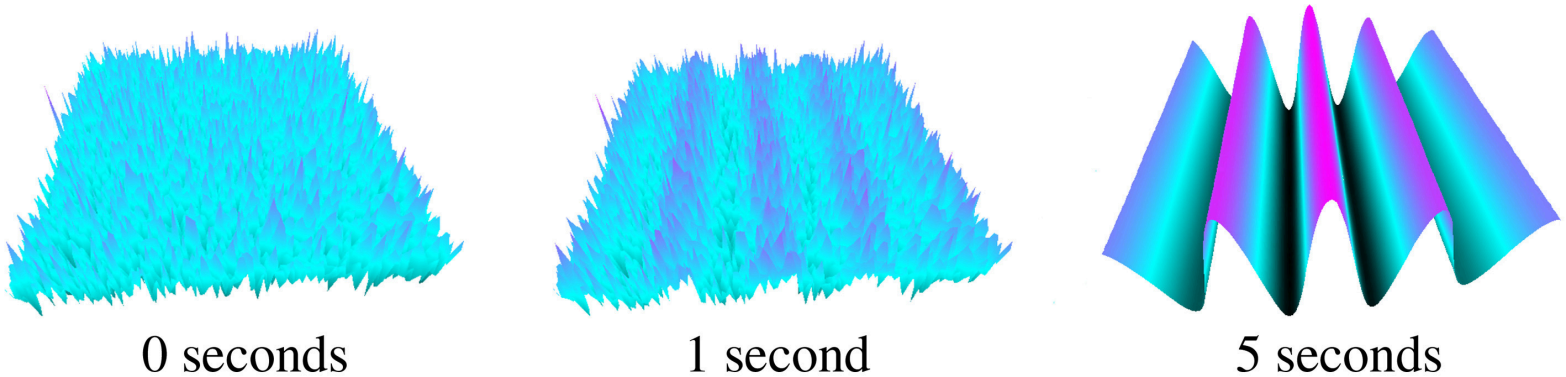
**Static Turing pattern emerging over 5 s**.

With *c* = 6364 the effective speed is infinite for the given *l* and *dt* properties. Figure 5 shows the simulation starting with random field potential noise. A pattern begins to emerge at 1 s and evolves into a temporally constant Turing pattern after approximately 5 s.

Dynamic Turing patterns emerging over time in the simulator with interface values:

$$\begin{array}{l} \text{showData}\;=\text{ 1 }\\ \text{endTime}\;=\;40\;\\ \text{dt}\;=\;0.00\text{5 }\\ \text{gamma}\;=\;0.\text{82 }\\ \text{eta}\;=\;1.0\;\\ \text{c}\;=\;10.0\;\\ \text{l}\;=\;10.0\\ \text{n}\;=\text{ 256 }\\ \text{V}0\;=\;\text{np}.\text{ones}\left(\left(\text{n},\text{n}\right)\right)*4.\text{1 }+\text{np}.\text{random}.\text{normal}\\ \;\left(0,0.1,\left(\text{n},\text{n}\right)\right)\\ \text{noiseVcont}\;=\;\text{None}\\ \text{Uexcite}\;=\;\text{np}.\text{zeros}\left(\left(\text{n},\text{n}\right)\right)\;\\ \text{I}\;=\;\text{np}.\text{ones}\left(\left(\text{n},\text{n}\right)\right)*2.0\;\\ \text{lins}\;=\;\text{np}.\text{linspace}\left(0,\;7*\text{np}.\text{pi},\;\text{n}\right)\;*-\text{1 }\\ \text{localStrong}\;=\;\text{np}.\text{linspace}\left(0,\;\text{np}.\text{pi},\;\text{n}\right)\;\\ \text{K}\;=\;\text{np}.\text{zeros}\left(\left(\text{n},\;\text{n}\right)\right)\;\\ \text{for}\;\text{i}\;\text{in}\;\text{range}\left(\text{n}\right):\;\\ \;\text{K}\left[:,\text{i}\right]\;=\;\text{np}.\text{sin}\left(\text{lins}\left[\text{i}\right]\right)\;*\;\text{np}.\text{sin}\left(\text{localStrong}\left[\text{i}\right]\right)\\ \text{for}\;\text{i}\;\text{in}\;\text{range}\left(\text{n}\right):\\ \;\text{K}\left[\text{i}\right]\;-=\;\text{np}.\text{sin}\left(\text{lins}\left[\text{i}\right]\right)\;*\;\text{np}.\text{sin}\left(\text{localStrong}\left[\text{i}\right]\right)\\ \text{def}\;\text{updateS}\left(\text{V}\right):\\ \;\text{return}\;1.0\;/\;\left(1+\text{np}.\text{exp}\left(-5.5*\left(\text{V}-3.0\right)\right)\right) \end{array}$$

**Figure 6:**
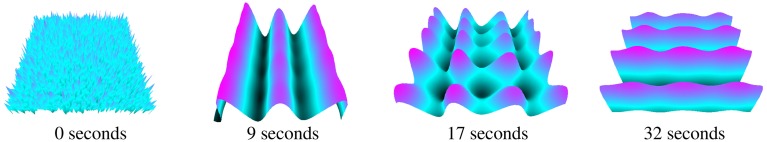
**Turing patterns in neural field activity forming over time during the same simulation**.

Figure 6 shows a typical simulation, given random starting field potential noise, with different Turing patterns materializing. Activity patterns form at varying intervals, generally every few seconds, throughout the simulation. The times in Figure 6 were chosen for clear displays of different (vertical, cone, and horizontal) patterns.

### 3.3. Finite spreading speed

**Figure 7:**
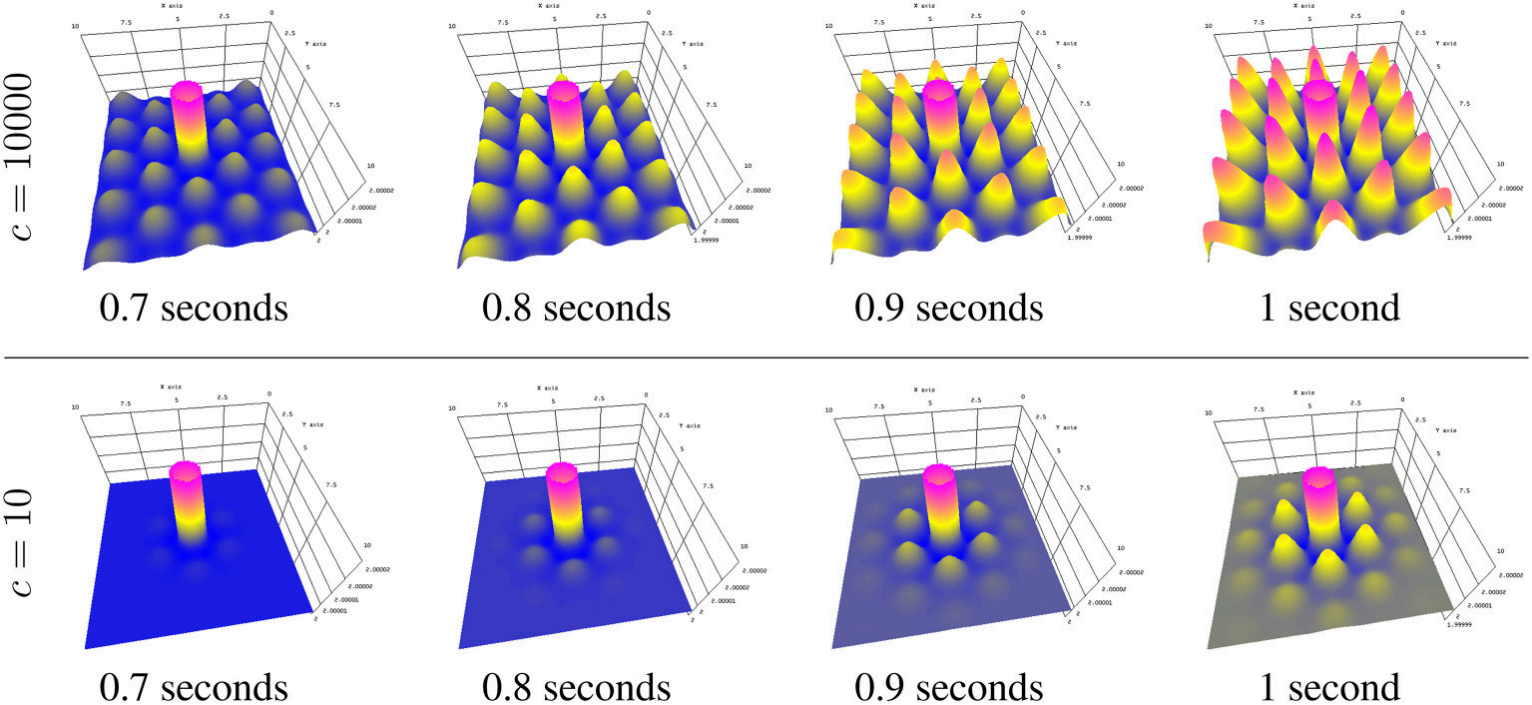
**Activity spread with large speed *c* (top) and small speed *c* (bottom)**.

Stimulating a neural population at a single location, as is done in typical physiological experiments applying external stimuli, the neural activity spreads in the population. Since finite transmission speed represents delayed spatial interaction in the population under study, it affects the spreading speed of activity (Hutt, 2007). If the transmission speed is infinite, the activity spreads diffusively involving the instantaneous activation at all spatial locations. Conversely, finite transmission speed delays the activity spread leading to a slowly-moving spreading front (Hutt and Atay, 2006; Hutt, 2009). Figure 7 shows numerical simulations for large (top row) and small speeds (bottom row), other parameters are identical.

The simulator allows the transmission speed to be examined closely in the visualization window by decreasing the maximum z axis value to be close to the original field value. This was done in Figure 7 by typing

y 2.00002 [Enter key]

after starting the simulator and before beginning the simulation.

The parameters to achieve the results in Figure 7 are

$$\begin{array}{l} \text{showData}\;=\text{ 1 }\\ \text{endTime}\;=\text{ 1 }\\ \text{dt}\;=\;0.00\text{4 }\\ \text{gamma}\;=\;1.0\;\\ \text{eta}\;=\;0.\text{35 }\\ \text{c}\;=\;10.0\;\\ \text{l}\;=\;10.0\\ \text{n}\;=\text{ 256 }\\ \text{V}0\;=\;\text{np}.\text{ones}\left(\left(\text{n},\text{n}\right)\right)*2.0\;\\ \text{noiseVcont}\;=\;\text{None}\;\\ \text{Uexcite}\;=\;\text{np}.\text{zeros}\left(\left(\text{n},\text{n}\right)\right)\;\\ \text{I}\;=\;2.0\;*\text{np}.\text{exp}\left(-\text{x}**2/0.04\right)/\left(0.04*\text{np}.\text{pi}\right)\;\\ \text{phi}\;=\;\text{np}.\text{pi}/\text{3 }\\ \text{K}\_\text{c}\;=\;10*\text{np}.\text{pi}/\text{l}\;\\ \text{K}\;=\;0.1*(\text{np}.\text{cos}\left(\text{K}\_\text{c}*\text{a}\right)\;+\;\backslash \;\\ \;\text{np}.\text{cos}(\text{k}\_\text{c}*(\text{a}*\text{np}.\text{cos}\left(\text{phi}\right)+\text{b}*\text{np}.\;\\ \;\text{sin}\left(\text{phi}\right))\text{)}+\;\backslash \\ \;\text{np}.\text{cos}(\text{K}\_\text{c}*(\text{a}*\text{np}.\text{cos}\left(\text{phi}*2\right)+\text{b}*\text{np}.\text{sin}\;\\ \;\left(\text{phi}*2\right))))\;*\;\backslash \\ \;\text{np}.\text{exp}\left(-\text{x}/10.0\right)*\left(\text{l}/\text{float}\left(\text{n}\right)\right)**2\\ \text{def}\;\text{updateS}\left(\text{V}\right):\;\\ \;\text{return}\;2.0\;/\;(1+\text{np}.\text{exp}(-5.5*\left(\text{V}-3.0\right))) \end{array}$$

where *c* is chosen according to the values given in Figure 7.

### 3.4. Extensions

**Figure 8:**
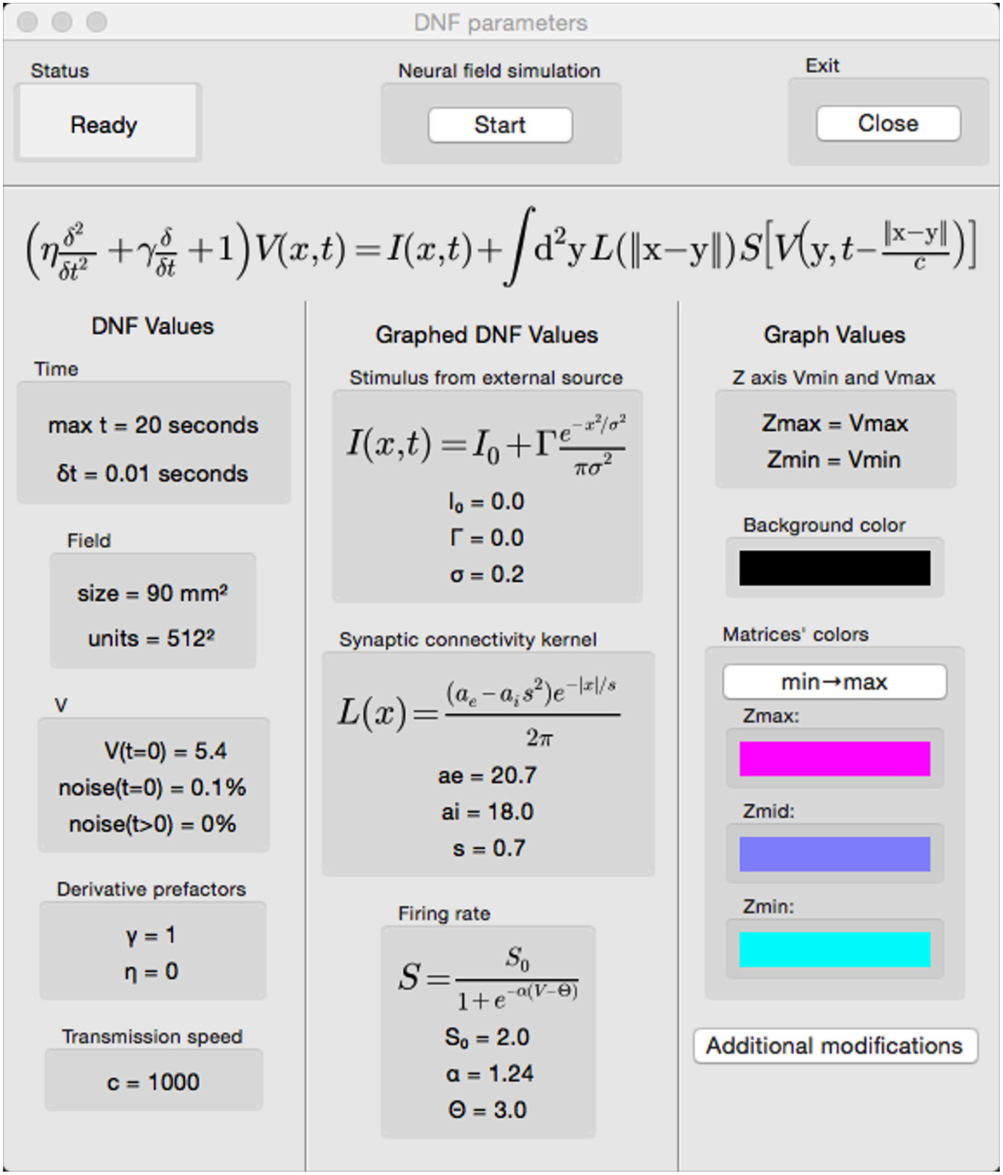
**A graphical interface for the simulator**.

The simulator, being open source, allows the tailoring of code to provide modifications and extensions. An example extension is the addition of a graphical interface to modify parameters and simulate neural fields. Figure 8 shows an example interface coded with the wxPython^6^ toolkit. Simulations are started, paused, continued, and started anew by clicking a button.

Neural field parameters can be modified prior to and during simulations by clicking on the appropriate area of the interface and completing a pop-up dialogue. Running simulations are automatically paused when a mouse hovers above parameter areas of the interface. There is a symbiosis among the show3D library discussed in Section 2.3 and the parameter selection interface. It is possible to view the external input stimulus, kernel, and firing rate in the GLFW window by adding a mouse event and hovering over these sections to automatically view the respective matrices. Viewing these elements while changing their parameters can help to fine-tune field parameters. Moving the mouse away from these areas unpauses paused simulations and the field potential matrix is shown in the GLFW window. Further synergy between the interface and show3D library is implemented with the option to alter graph values from the interface where z axis limits and colors can be selected.

## 4. Discussion

The Neural Field Simulator and its dependencies are cross-platform. However, the simulator interacts with graphics hardware using system-specific drivers which can result in problems on some operating systems. The graphical user interface in Section 3.4 is an example of this where the cross platform wxPython toolkit uses OpenGL to draw to the screen. The show3D library likewise uses OpenGL to interact with GPU. The graphical user interface and show3D library function symbiotically on Mac systems via the Apple Graphics Library. Conversely, on other operating systems such as Linux and Windows, unfortunately the separate utilization of OpenGL causes the simulator to crash. To this end, the current version of the simulator is released without the addition of extensions in order to operate properly on every major operating system. Nevertheless, a graphical interface can be a good choice with an appropriate single operating system.

Apart from the software implementation, in future work some model assumptions can be released. The square geometry can be recast easily to a rectangular geometry, whereas more general geometries (e.g., the impressive implementation work in *The Virtual Brain* Sanz Leon et al., 2015) will take more numerical effort. Periodic boundary conditions guarantee the simple application of the FFT, effective implementations of other boundary conditions like Dirichlet conditions [*V*(**z**, *t*) = const, **z** ∈ ∂Ω] will demand some implementation changes in the spatial integral computation. Such modifications may still retain the fundamental implementation of the FFT. In contrast, rejecting the homogeneity hypothesis of spatial interactions, i.e., *K* = *K*(**x, y**) ≠ *K*(**x** − **y**), abolishes the convolution structure and slows down the numerical simulation, cf. Appendix I.

Future work will extend the NFM model to multiple equations to render the model even more biologically plausible. In addition, an extension of the implementation to a mixture of constant and space-dependent delays as considered by Veltz and Faugeras (2011) will be interesting.

### Conflict of interest statement

The authors declare that the research was conducted in the absence of any commercial or financial relationships that could be construed as a potential conflict of interest.

## Footnotes

^1^https://gforge.inria.fr/projects/nfsimulator/.

^2^http://nfsimulator.gforge.inria.fr.

^3^https://senselab.med.yale.edu/ModelDB/showModel.cshtml?model=184479.

^4^http://show3d.gforge.inria.fr/index.html.

^5^https://gforge.inria.fr/projects/show3d/.

^6^http://www.wxpython.org.

## Appendix i

### Heterogeneous neural fields

Heterogeneous neural fields have attracted increased attention in recent years (Bressloff, 2001; Qubbaj and Jirsa, 2007; Brackley and Turner, 2009; Schmidt et al., 2009; Coombes et al., 2012; beim Graben and Hutt, 2014) since they have been found in biological neural networks such as the prefrontal cortex (Rosenkilde, 1979; Wang et al., 2006) and visual cortex (Demeulemeester et al., 1988). In order to study the neural population activity in such systems, the present implementation could be extended along the following mathematical reasoning. The integral in Equation (1) may be re-written as

$$\begin{array}{l} \int_{\Omega }{\text{d}}^{2}y\;K(x,y)\;S\left[V\left(y,t-\frac{∥x-y∥}{c}\right)\right]\\ =\int_{\Omega }{\text{d}}^{2}y\int_{-\infty }^{\infty }d\tau K(x,y)\delta (\tau -t+\frac{∥x-y∥}{c})\;S\left[V\left(y,\tau \right)\right]\\ =\int_{\Omega }{\text{d}}^{2}y\int_{0}^{\tau_{max}}dTD(x-y,T)\;R(x,y,t-T) \end{array}$$

with *D*(**x**, *t*) = δ(∥ **x** ∥ ∕*c* − *t*), *R*(**x, y**, *t*) = *K*(**x, y**)*S*[*V*(**y**, *t*)]. This integral still has a spatial convolution structure; however, it is not perfect since *R* includes the spatial location **x**. The numerical simulation of the integral consequently involves $n^{4}n_{rings}$ summands and the numerical implementation is slower than in the homogeneous case. The formulation of heterogeneous neural fields is not implemented yet, but it will be part of a future update.

## Appendix ii

**Table A1:** **Keyboard keys and their actions**.

**Key**	**Action**
2	Interpolate between min and max graph value colors
3	Interpolate between min-mid and mid-max graph value colors
↑	Rotate the field up
↓	Rotate the field down
←	Rotate the field left
→	Rotate the field right
a	Modulate minimum graph value color
b	Modulate background graph color
d	Move the field down
e	Move the field up
Esc	Exit simulation
f	Move the field right
g	Change number of axes lines
i	Save an image
j	Set min and max z axis limits to min and max field values
k	Change color distribution to a higher range
l	Change color distribution to a reduced range
m	Set minimum z axis limit to minimum field value
n	Change minimum z axis limit
o	Equally distribute color range
p	Pause and resume simulation
*pg up*	Zoom in
*pg down*	Zoom out
q	Modulate middle graph value color
s	Move the field left
t	Change axis text size on Mac systems
u	Set maximum z axis limit to maximum field value
v	Begin and end video recording
y	Change maximum z axis limit
z	Modulate maximum graph value color
